# Bibliography

**Published:** 1902-07-15

**Authors:** 


### BIBLIOGRAPHY.

A NEW edition of Abbott's Bacteriology has just come from the press of Leo Brothers & Co. This is the sixth edition of this work in the ten years of its publication. This fact will be more covincing of its worth than any comment of ours. Dr. Abbott is Professor of Hygiene in the University of Pennsylvania, and is an authority in this subject, as he is an original investigator in the field of biological bacteriology. The present work is in one volume of over six hundred pages and covers the field of bacteriology in a technical way, making it a valuable text book for students. At the same time the book has much of interest to the medical practitioner, because it brings up to date the more recent results of investigations of such diseases as epidemic cerebro-spinal meningitis, dysentery, tuberculosis and the later views on immunity. As a technical work we know of none better for dental students or practitioners who may desire a better knowledge of the science of bacteriology. The work has no particular dental application, except the chapter on disinfection and that on infection and immunity. We can cordially recommend the work to any one wishing a brief but up-to-date comprehensive statement of the principles of bacteriology.

